# Hypothesis-Generating Magnetic Resonance Imaging (MRI) Observation of Maisonneuve Fractures With Discontinuous Interosseous Membrane Injury: A Three-Case Series

**DOI:** 10.7759/cureus.99427

**Published:** 2025-12-16

**Authors:** Makoto Sato, Tadashi Kimura, Mitsuru Saito, Takeshi Fukuda, Makoto Kubota

**Affiliations:** 1 Department of Orthopaedic Surgery, The Jikei University School of Medicine, Tokyo, JPN; 2 Department of Radiology, The Jikei University School of Medicine, Tokyo, JPN

**Keywords:** ankle fracture, haraguchi classification, interosseous membrane, lauge–hansen classification, maisonneuve fracture

## Abstract

Maisonneuve fractures, proximal fibular fractures with concomitant distal tibiofibular disruption and either medial malleolus fracture or deltoid ligament injury, represent ~5% of ankle fractures and are uncommon pronation-external‑rotation ankle injuries whose interosseous‑membrane (IOM) damage is widely believed to be continuous from the ankle to the proximal fibular fracture. However, this hypothesis was proposed in the literature prior to the widespread use of magnetic resonance imaging (MRI). This study aimed to describe MRI patterns of IOM injury in Maisonneuve fractures from a small three-case series and to reconsider the traditional assumption of a full‑length IOM rupture. We reviewed MRI scans and defined IOM disruption as loss of the continuous low‑signal band between the tibial and fibular cortices with corresponding high signal on short tau inversion recovery (STIR). Distal (A), proximal (B), and intact mid‑segment (C) lengths were measured on coronal images. All cases demonstrated distal and proximal IOM injury with a preserved mid‑segment. Recognition of this discontinuous injury pattern on MRI challenges the concept of full‑length IOM rupture.

## Introduction

Ankle fractures are common, accounting for approximately 9% of all fractures and showing a wide variety of fracture types [1]. The Maisonneuve fracture, first described by Jacques Gilles Maisonneuve in 1840, is relatively rare, accounting for approximately 5% of all ankle fractures [2]. It is characterized by a proximal fibular fracture accompanied by distal tibiofibular ligament injury and either a medial malleolus fracture or deltoid ligament injury. This fracture is easily missed or diagnosed late, so the actual prevalence may be underestimated [3]. Maisonneuve fractures are often reported to be caused by high-energy trauma [4] and are notable for extensive damage to the interosseous membrane (IOM) [5]. IOM is an important stabilizer of the ankle syndesmosis, which extends transversely between the tibia and fibula and has a continuation that extends proximally between the tibia and fibula [6]. Previous studies have shown that the IOM is torn continuously from the syndesmosis to the level of the proximal fibular fracture [7,8]. However, it was difficult to make quantitative evaluations during the acute phase before the era of MRI because the IOM cannot be seen in a plain radiograph or computed tomography (CT) scan, and this hypothesis was first put forward in the literature before MRI scans became widely available. Furthermore, a prior report has described MRI images of a discontinuous pattern of IOM involvement in Maisonneuve fractures [9]. While the exact injury mechanism was not determined for each case in this report, we focus on some cases caused by low-energy forces and with mild symptoms of swelling and pain in the lower leg. We doubted whether this hypothesis would apply to such low-energy cases. The purpose of this study was to evaluate the actual extent of IOM damage and whether IOM injury in Maisonneuve fractures consistently demonstrates a continuous rupture.

## Case presentation

**Figure 1:**
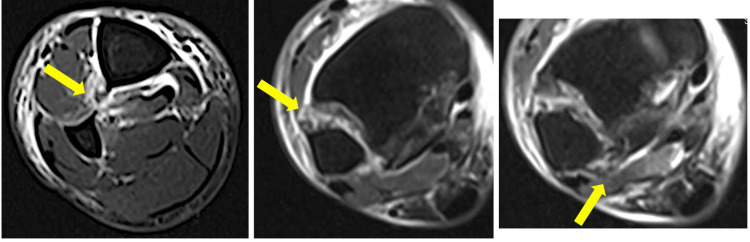
An axial short tau inversion recovery image showing an interosseous membrane injury and the distal tibiofibular ligament injury. Interosseous membrane injury was defined as a disruption of the continuous low-signal band between the tibial cortex and fibular cortex, accompanied by high signal intensity reflecting bleeding or edema at the same site (left arrow). The distal tibiofibular ligament was defined as damaged if high signal intensity was observed in the ligament on coronal STIR images (the middle arrow shows the anterior inferior tibiofibular ligament). The right arrow shows the posterior inferior tibiofibular ligament.

The injury to the IOM and distal tibiofibular ligament was evaluated on magnetic resonance images (MRI) scans of the lower leg obtained within seven days after the injury. MRI was performed on a clinical 1.5 tesla MRI unit with a multichannel rare coil (TOSHIBA EXCELART VANTAGE MRT-2003/P2 [Tokyo, Japan]). Based on prior research concerning MRI findings of interosseous membrane rupture [9], an IOM injury was defined on axial short tau inversion recovery (STIR) images as a disruption of the continuous low-signal band between the tibial cortex and fibular cortex, accompanied by high signal intensity reflecting bleeding or edema at the same site (Figure 1). This case series included all consecutive patients with Maisonneuve fractures who presented to and were managed at our institution during the study period.

**Figure 2:**
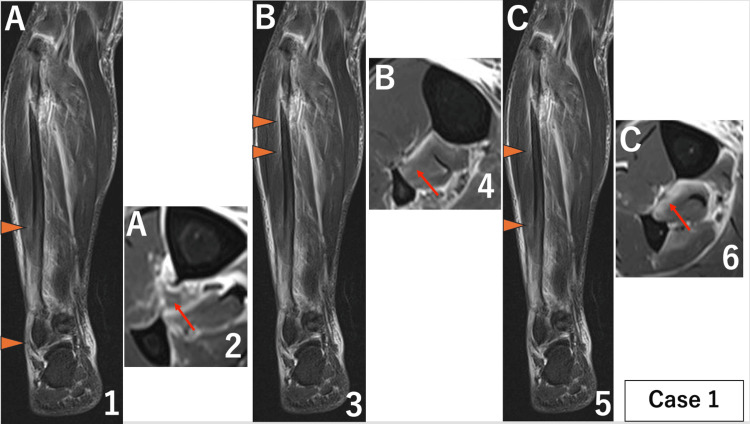
Magnetic resonance images (MRI) of the right lower leg for case 1. When a disruption of the low-signal band from the tibial cortex to the fibular cortex was identified as an interosseous membrane injury on axial images, the extent of the injury was measured on coronal images. Parts where the interosseous membrane was intact were also measured on coronal images, (A) part of distal injury. ① Coronal image of MRI. Orange arrows show the extent of the injury measured on coronal images was 119 mm. ② Axial image of MRI. The red arrow shows interosseous membrane injury. (B) Part of proximal injury. ③ Coronal image of MRI. Orange arrows show the extent of the injury measured on coronal images was 28 mm. ④ Axial image of MRI. The red arrow shows interosseous membrane injury. (C) Non-injured part. ⑤ Coronal image of MRI. Orange arrows show the extent of the injury measured on coronal images was 79 mm. ⑥ Axial image of MRI. The red arrow shows that the interosseous membrane is intact. The primary finding was that the central interosseous membrane remained intact, differing from previous studies reporting damage to the entire length of the interosseous membrane.

The length of the injury was measured on coronal images and defined as follows: A, the distal injury part (extent of injury measured proximally from the distal end of the tibia); B, the proximal injury part (extent of injury measured distally from the lower end of the fibular fracture); and C, the non-injured part (distance between A and B) (Figure 2).

The distal tibiofibular ligament was defined as damaged if high signal intensity was observed in the ligament on coronal STIR images (Figure 1). Interpretation and measurements were performed by three orthopedic surgeons and one radiologist specializing in bone and soft tissue. Any disagreements were discussed until a consensus was reached.

The following three cases were examined (Table 1).

**Table 1:** Case summary AITFL: anterior inferior tibiofibular ligament; PITFL: posterior inferior tibiofibular ligament; IOM: interosseous membrane; ROM: range of motion; NV: neurovascular; MSK: musculoskeletal.

Field	Case 1	Case 2	Case 3
Age (years)	53	44	25
Sex	Male	Male	Male
Side (Right/Left)	Right	Right	Left
Occupation / Activity level	Office worker	Circus performer	Office worker
Relevant medical history (e.g., osteoporosis, prior injuries)	None	None	None
Family history (MSK/connective tissue)	None	None	None
Injury mechanism (narrative)	Fell during a golf swing with external rotation of the right ankle.	Fell from a unicycle, landing with the right ankle in external rotation.	Missed a step and fell with external rotation of the left ankle.
Biomechanical interpretation (force direction / rotation) in the Lauge–Hansen classification.	Pronation-external rotation (PER) type	Pronation-external rotation (PER) type	Pronation-external rotation (PER) type
Tenderness	Ankle, Fibular head	Ankle, Fibular head	Ankle, Fibular head
Deformity	None	None	None
Range of motion	Limited	Limited	Limited
Swelling	Mild	Strong	Mild
Skin integrity	Intact	Blister	Intact
Neurovascular status	No abnormalities	No abnormalities	No abnormalities
Weight-bearing ability at presentation	Impossible	Impossible	Impossible
Proximal fibular fracture on X-ray/CT	Yes	Yes	Yes
Deltoid / Medial malleolus fracture on X-ray/CT	No	Yes	Yes
Posterior malleolus fracture on X-ray/CT	Yes	No	No
Syndesmosis (anterior widening) on X-ray/CT	Yes	Yes	Yes
Treatment	Operation	Operation	Operation
Follow-up	1.0Y	1.5Y	1.5Y

This case series included all consecutive patients with Maisonneuve fractures who presented to and were managed at our institution during the study period.

Case 1

**Figure 3:**
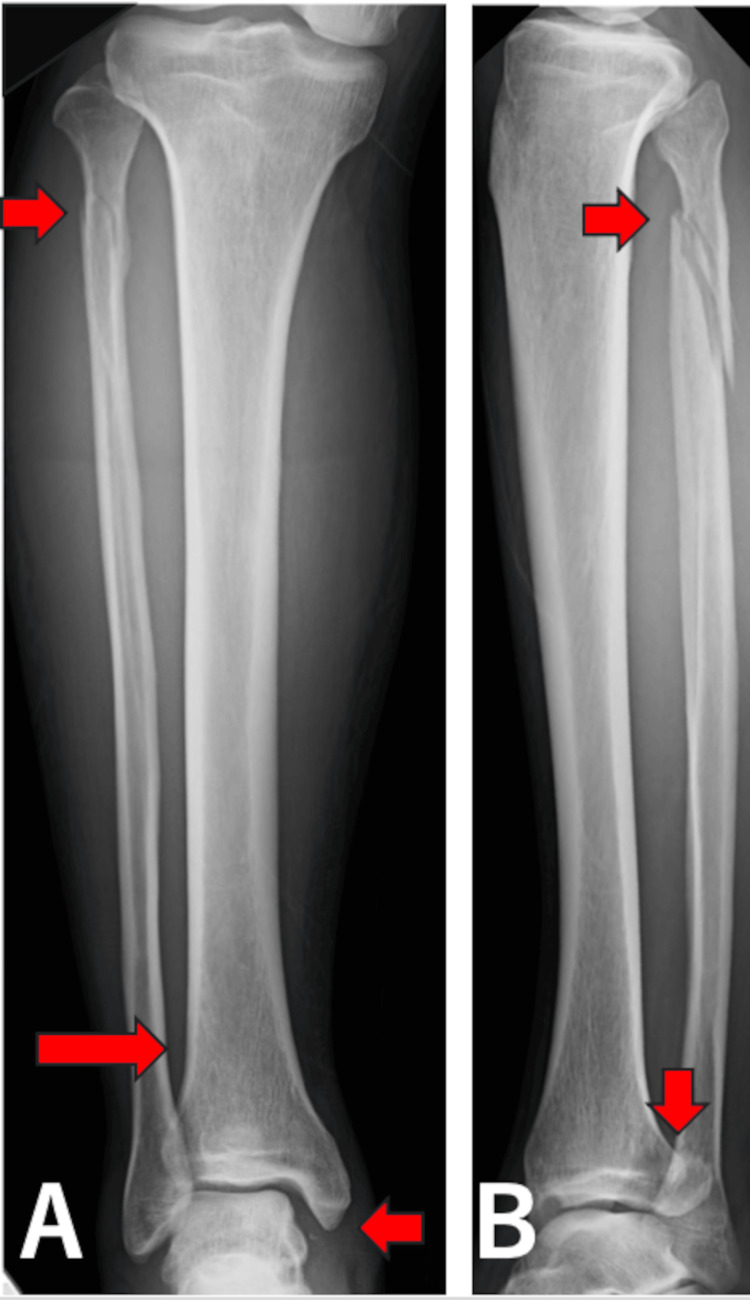
Anteroposterior (A) and lateral (B) plain radiographs for case 1. Red arrows show medial clear space widening, anterior widening of the tibiofibular area, a posterior malleolus fracture, and an oblique fracture of the proximal fibula.

**Figure 4:**
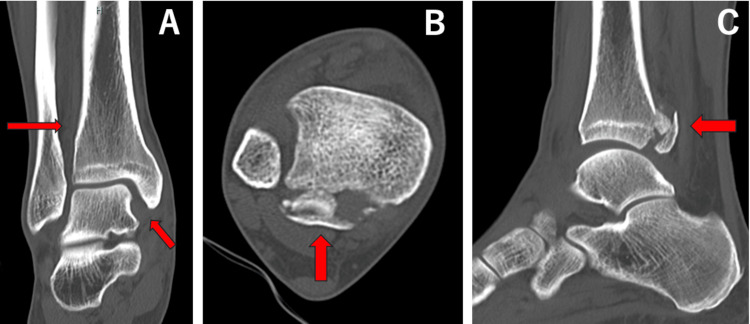
Coronal (A), axial (B), and sagittal (C) images of computed tomography for case 1. Red arrows show medial clear space widening, anterior widening of the tibiofibular area, and a posterior malleolus fracture.

A 53-year-old man sustained an injury while playing golf. During a swing on a slope, he fell with his right ankle in an externally rotated position. He visited our outpatient clinic the day after the injury. On initial examination, in addition to pain in the ankle, tenderness was observed at the fibular head. Plain radiographs and computed tomography (CT) images were taken on the initial visit and showed widening of the medial clear space and an anterior tibiofibular area, along with a posterior malleolus fracture and an oblique fracture of the proximal fibula (Figures 3, 4).

Based on physical examination findings and imaging findings, a Maisonneuve fracture was diagnosed on the day of the initial visit. An MRI was performed three days after the initial presentation. The surgery was performed seven days after the initial presentation. Following a 12-month follow-up period, no residual ankle instability or pain was observed.

Case 2

**Figure 5:**
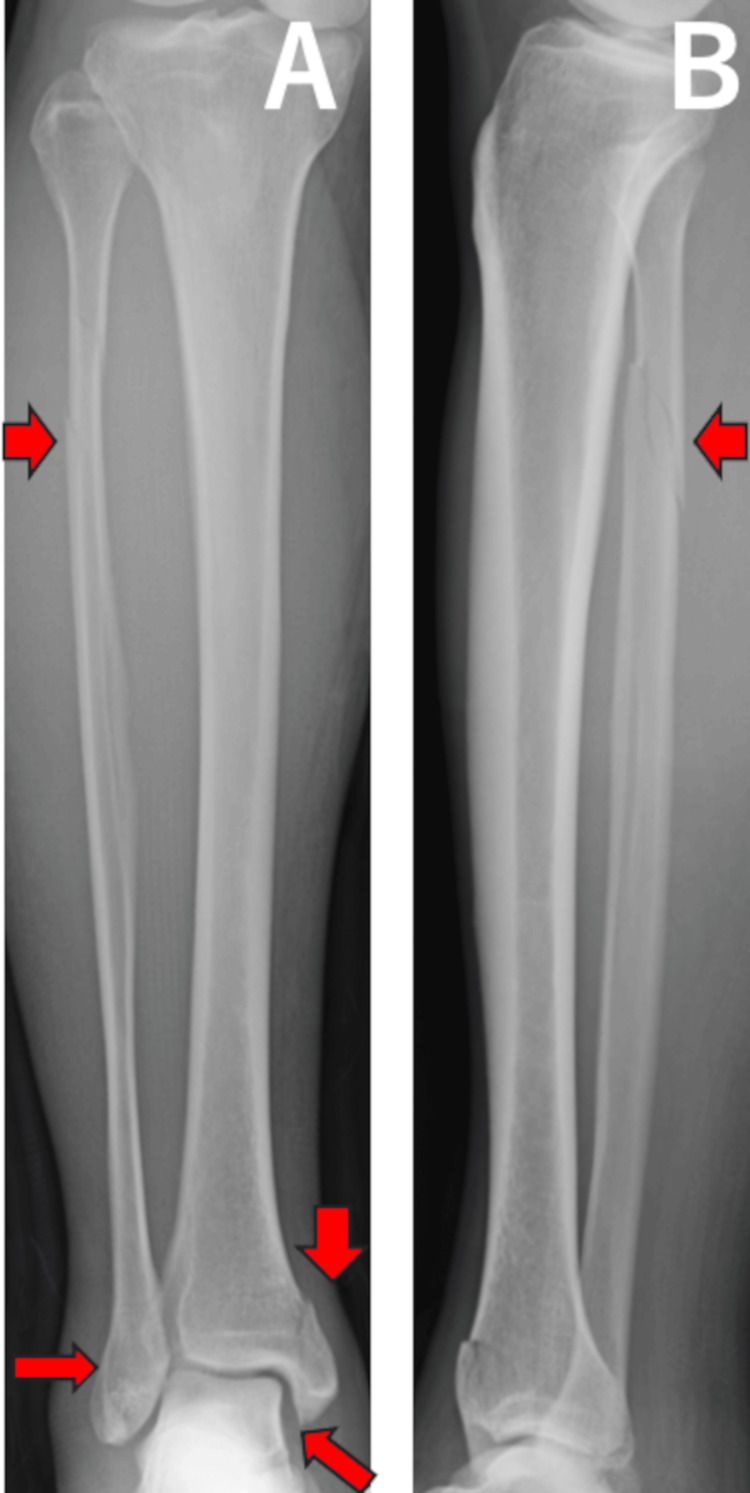
Anteroposterior (A) and lateral (B) plain radiographs for case 2. Red arrows show medial clear space widening, a medial malleolus fracture, and an oblique fracture of the proximal fibula with lateral displacement of the talus within the ankle joint.

**Figure 6:**
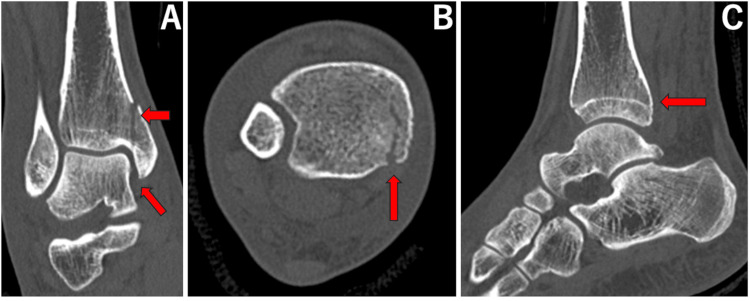
Coronal (A), axial (B), and sagittal (C) images of computed tomography for case 2. Red arrows show medial clear space widening and a medial malleolus fracture and a normal posterior malleolus.

A 44-year-old man fell while riding a unicycle, landing with his right ankle in an externally rotated position. He visited our outpatient clinic four days after the injury. On initial examination, tenderness was present at the fibular head and ankle. Plain radiographs and CT images were taken on the initial visit and showed widening of the medial clear space, a medial malleolus fracture, and an oblique fracture of the proximal fibula (Figures 5, 6).

Based on physical examination findings and imaging findings, a Maisonneuve fracture was diagnosed on the day of the initial visit. MRI was performed four days after the initial presentation. The surgery was performed seven days after the initial presentation. Following an 18-month follow-up period, no residual ankle instability or pain was observed.

Case 3

**Figure 7:**
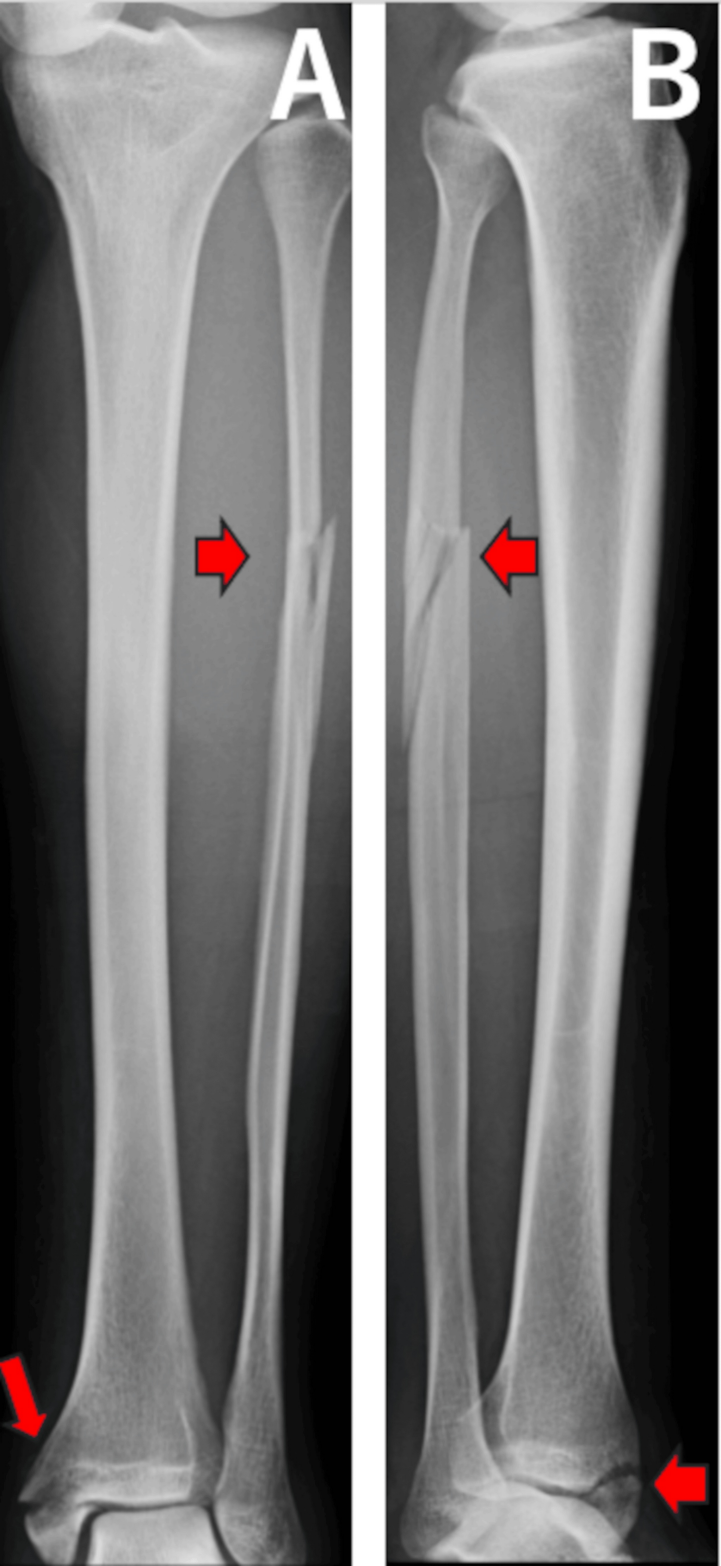
Anteroposterior(A) and lateral (B) plain radiographs for case 3. Red arrows show a medial malleolus fracture and an oblique fracture of the proximal fibula.

**Figure 8:**
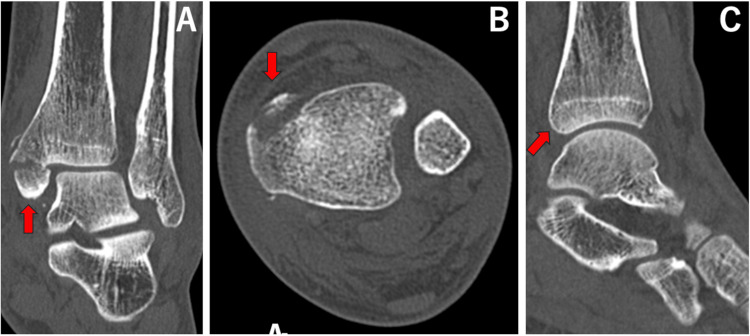
Coronal (A), axial (B), and sagittal (C) images of computed tomography for case 3. Red arrows show a medial malleolus fracture and a normal posterior malleolus.

A 25-year-old man missed a step on a staircase and fell, causing his left ankle to rotate externally. He visited our outpatient clinic the day after the injury. On initial examination, in addition to pain in the ankle, there was tenderness along the fibular shaft. Plain radiographs and CT images were taken on the initial visit and showed a fracture of the medial malleolus and an oblique fracture of the proximal fibula, with the talus displaced laterally within the ankle joint (Figures 7, 8).

Based on physical examination findings and imaging findings, a Maisonneuve fracture was diagnosed on the day of the initial visit. An MRI was performed five days after the initial presentation. The surgery was performed eight days after the initial presentation. Following an 18-month follow-up period, no residual ankle instability or pain was observed.

**Figure 9:**
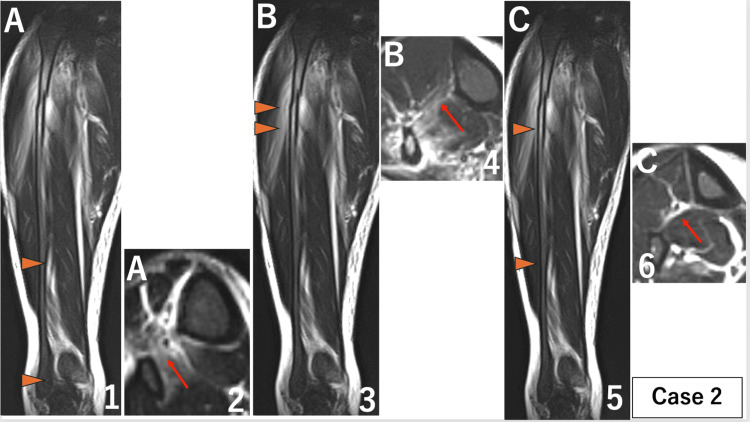
MRI of the lower right leg for case 2. When a disruption of the low-signal band from the tibial cortex to the fibular cortex was identified as an interosseous membrane injury on axial images, the extent of the injury was measured on coronal images. Parts where the interosseous membrane was intact were also measured on coronal images. (A) Part of distal injury: ① Coronal image of MRI. Orange arrows show the extent of the injury measured on coronal images was 102 mm. ② Axial image of MRI. The red arrow shows interosseous membrane injury. (B) Part of proximal injury: ③ Coronal image of MRI. Orange arrows show the extent of the injury measured on coronal images was 22 mm. ④ Axial image of MRI. The red arrow shows interosseous membrane injury. (C) Non-injured part: ⑤ Coronal image of MRI. Orange arrows show the extent of the non-injured part in the middle 135 mm section. ⑥ Axial image of MRI. The red arrow shows that the interosseous membrane is intact. The primary finding was that the central interosseous membrane remained intact, differing from previous studies reporting damage to the entire length of the interosseous membrane.

**Figure 10:**
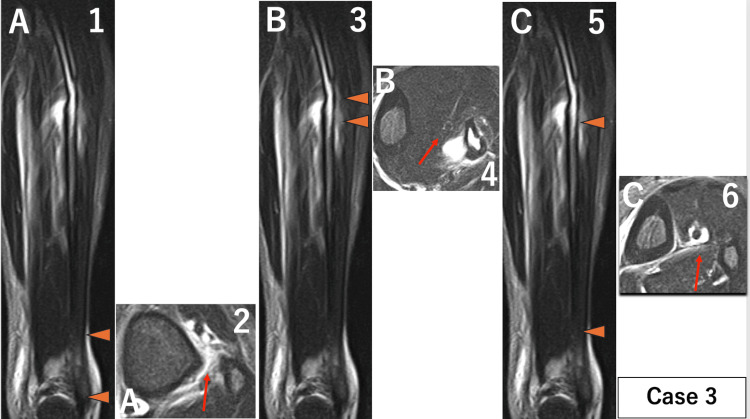
MRI of the left lower leg for case 3. When a disruption of the low-signal band from the tibial cortex to the fibular cortex was identified as an interosseous membrane injury on axial images, the extent of the injury was measured on coronal images. Parts where the interosseous membrane was intact were also measured on coronal images. (A) Part of distal injury: ① Coronal image of MRI. Orange arrows show the extent of the injury measured on coronal images was 38 mm. ② Axial image of MRI. The red arrow shows interosseous membrane injury. (B) Part of proximal injury: ③ Coronal image of MRI. Orange arrows show the extent of the injury measured on coronal images was 11 mm. ④ Axial image of MRI. The red arrow shows interosseous membrane injury. (C) Non-injured part: ⑤ Coronal image of MRI. Orange arrows show the extent of the non-injured part in the middle 184 mm section. ⑥ Axial image of MRI. The red arrow shows that the interosseous membrane is intact. The primary finding was that the central interosseous membrane remained intact, differing from previous studies reporting damage to the entire length of the interosseous membrane.

The extent of injury to the IOM in each case was as follows. Case 1: A 119 mm, B 28 mm, and C 79 mm (Figure 2). Case 2: A 102 mm, B 22 mm, and C 135 mm (Figure 9). Case 3: A 38 mm, B 11 mm, and C 184 mm (Figure 10).

The anterior inferior tibiofibular ligament (AITFL) was damaged in all cases, and the posterior inferior tibiofibular ligament (PITFL) remained intact in two cases. There was no injury to the IOM proximal to the fibular fracture site in any of the cases (Table 2).

**Table 2:** Distances of the A, B, and C regions and tibiofibular ligament injuries for each case AITFL: anterior inferior tibiofibular ligament; PITFL: posterior inferior tibiofibular ligament; “+” means damaged and “-” means intact.

Case	A Distal Injury (mm)	B Proximal Injury (mm)	C Non-injured Area (mm)	Ligament Injury	
				AITFL	PITFL
1	119	28	79	+	+
2	102	22	135	+	−
3	38	11	184	+	−

## Discussion

In a Maisonneuve fracture, a proximal fibular fracture is observed along with diastasis of the tibiofibular joint with either a rupture of the deltoid ligament or a medial malleolus fracture. These injuries are considered to involve a strong external rotational force and are classified as a subtype of the pronation-external rotation (PER) type sequences in the Lauge-Hansen classification, in which medial restraints fail first, followed by anterior syndesmotic disruption and interosseous membrane (IOM) injury. According to this classification, external rotational force ruptures the IOM and is transmitted proximally to the fibula, causing a fracture and continuous damage to the IOM from the distal tibiofibular ligament to the proximal site of the fibular fracture [7,8]. Haraguchi’s mechanism-based reinterpretation, however, emphasizes that the resultant ankle fracture pattern is governed by the combination of the external-rotation moment and the abduction moment acting on a pronated foot, distinguishing abduction (ABD) from abduction plus external rotation (ABD+ER) injury vectors. In cadaver experiments, increasing a laterally directed load at the foot (thereby augmenting the abduction moment) shifted injuries toward high fibular fractures with a reversed obliquity, whereas lesser abduction loads produced short oblique distal fibular fractures, both occurring with the foot pronated [10]. If the injury mechanism of the Maisonneuve fracture is similar to PER or ABD+ER, where a proximal fibular fracture results from abduction force at the level of the ankle, significant widening of the distal tibiofibular joint would be required, with the fibula being pressed against the talus. This sequence of events, namely, continuous IOM rupture followed by a proximal fibular fracture, would not occur without such substantial widening. If continuous IOM damage is present from the ankle to the proximal fibula, severe pain and swelling in the lower leg would be expected; however, such symptoms are often absent in patients with these injuries. Furthermore, it is known that proximal fibular fractures are frequently missed on initial examination [11], likely because of the mild symptoms in the lower leg, including at the fibular fracture site. The IOM securely connects the tibia and fibula, contributing to their stability and withstanding significant tensile forces [12]. The distal part of the IOM is strong and tightly tensioned, but the proximal two-thirds have S-shaped slack [13], such that resistance to rotational forces varies by part [14]. When an external rotational force is applied to the fibula, the distal part of the IOM retains its shape, while the proximal part elongates and rotates externally, dissipating the force [14]. Based on clinical experience and these anatomical characteristics, we questioned the conventional belief that IOM is damaged along its entire length.

The characteristic injuries seen in Maisonneuve fractures include an oblique fracture of the proximal one-third of the fibula, extending from the anteromedial to the posterolateral direction, and a complete rupture of the AITFL [11]. Fibular fractures and AITFL injuries were also typical findings in our patients. He et al. reported that medial malleolus fractures or deltoid ligament injuries were almost inevitable in patients with a Maisonneuve fracture, with only one of 41 cases (2.4%) lacking such damage, while the PITFL was intact in 15% of cases [11]. This may reflect the fact that the PITFL is stronger than the anterior ligament [5,15] and serves as a hinge during external rotation injuries. Two of our three cases showed no damage to the PITFL, while the remaining case had an avulsion fracture at the ligament attachment site. Moreover, the posterior malleolar fractures associated with this injury are reported to occur significantly more often than ligament ruptures, with the extent of damage rarely exceeding one-quarter of the articular surface [16].

Although all our cases resulted from relatively small external forces, it became apparent that the middle part of the IOM may remain intact in this type of injury even if it is damaged at both the fibular fracture site and the distal tibiofibular ligament. Similar findings have been described in a previous MRI study. Compared with He et al. [9], our study adopted a similar MRI acquisition window and the operational definition of interosseous membrane (IOM) disruption on axial fluid-sensitive sequences, namely, loss of the continuous low-signal band with concordant hyperintensity. In addition, by incorporating segmental measurements on coronal images (A/B/C), we believe we obtained complementary information that refines anatomical mapping of IOM involvement in acute Maisonneuve fractures. He reported universal AITFL rupture and reproducible IOM phenotypes: 13/15 patients exhibited segmental IOM disruption, a distal tear, and a second tear near the fibular fracture, separated by an intact mid-segment [9]. Our series converges closely with these observations. All three acute cases demonstrated distal and proximal IOM disruption separated by a spared mid-segment, universal AITFL injury, and no IOM extension proximal to the fibular fracture on early MRI. We hypothesize that the following mechanism may explain this phenomenon.

**Figure 11:**
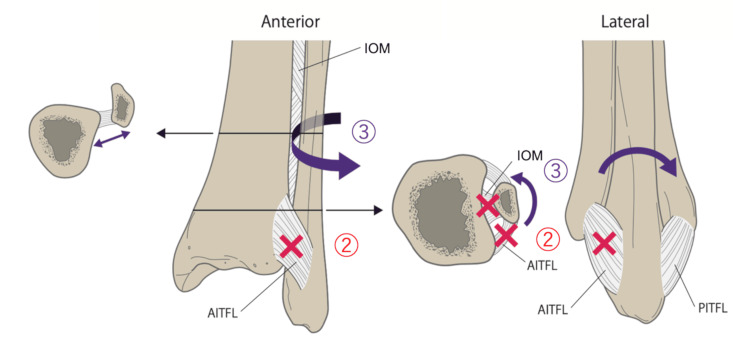
The area marked “2” in the diagram shows that as the talus rotates externally, forces open the distal tibiofibular joint from the anterior side, leading to ruptures of the AITFL and distal IOM. The area marked “3” in the diagram shows that with the application of additional rotational force, the fibula undergoes external rotation around the PITFL, acting as a hinge. When the rotational force reaches the proximal fibula, the middle interosseous membrane elongates and avoids rupture. AITFL: anterior inferior tibiofibular ligament; IOM: interosseous membrane; PITFL: posterior inferior tibiofibular ligament. The image is created by the author.

First, abduction and external rotation forces applied to the ankle disrupt the medial structures, resulting in either a medial malleolus fracture or a deltoid ligament injury. Second, as the talus rotates externally, it forces open the distal tibiofibular joint from the anterior side, leading to ruptures of the AITFL and distal IOM. Third, with the application of additional rotational force, the fibula undergoes external rotation around the PITFL, acting as a hinge [16]. If the force exceeds the tolerance of the PITFL, it often results in a posterior malleolar avulsion fracture or ligament rupture in some cases. Even when the rotational force reaches the proximal fibula, the middle IOM elongates and avoids rupture. Finally, the proximal fibula fracture occurs because of its strong connection to the tibia via the proximal tibiofibular ligament. Simultaneously, the IOM near the fracture site is also damaged (Figure 11 [17]).

This study has important limitations. First, it is a very small, homogeneous series (three men aged 25-53 years) with low-energy mechanisms; external validity is therefore limited. Second, we lacked a comparison cohort, including high-energy injuries, precluding statements about mechanism-dependent differences. Third, there was no surgical or histologic correlation with IOM status. Fourth, formal inter- and intra-observer reliability metrics for the MRI measurements were not obtained. Fifth, MRI interpretation is susceptible to artifacts and resolution constraints, and acute edema may mimic fiber disruption in the early phase, which leads to potentially overestimating the extent or frequency of IOM injury. Without follow-up MRI or surgical/histologic correlation, misclassification bias cannot be excluded, and our inferences should be regarded as hypothesis-generating. All these factors constrain the strength of inference and should be addressed in prospective studies with larger, more diverse samples and predefined reliability assessments. Also, this mechanistic model should be validated in instrumented cadaveric studies that systematically vary the abduction component.

## Conclusions

Early MRI in three acute Maisonneuve fractures demonstrated a discontinuous IOM injury pattern characterized by distal and proximal disruption separated by a spared mid-segment. These observations generate a hypothesis that challenges previous research. Larger and comparative studies with formal reliability testing and cadaveric studies are warranted to determine how often this pattern occurs.
